# Characterization of the Rotating Exercise Quantification System (REQS), a novel Drosophila exercise quantification apparatus

**DOI:** 10.1371/journal.pone.0185090

**Published:** 2017-10-10

**Authors:** Louis Patrick Watanabe, Nicole C. Riddle

**Affiliations:** Department of Biology, The University of Alabama at Birmingham, Birmingham, Alabama, United States of America; Lancaster University, UNITED KINGDOM

## Abstract

Obesity is a disease that has reached epidemic proportions in the United States and has prompted international legislation in an attempt to curtail its prevalence. Despite the fact that one of the most prescribed treatment options for obesity is exercise, the genetic mechanisms underlying exercise response in individuals are still largely unknown. The fruit fly *Drosophila melanogaster* is a promising new model for studying exercise genetics. Currently, the lack of an accurate method to quantify the amount of exercise performed by the animals is limiting the utility of the Drosophila model for exercise genetics research. To address this limitation, we developed the Rotational Exercise Quantification System (REQS), a novel apparatus that is able to simultaneously induce exercise in flies while recording their activity levels. Thus, the REQS provides a method to standardize Drosophila exercise and ensure that all animals irrespective of genotype and sex experience the same level of exercise. Here, we provide a basic characterization of the REQS, validate its measurements using video-tracking technology, illustrate its potential use by presenting a comparison of two different exercise regimes, and demonstrate that it can be used to detect genotype-dependent variation in activity levels.

## Introduction

Many studies have demonstrated the beneficial effects of exercise and physical activity on human health [1–3]. For example, upon initiation of regular exercise, improvements in muscle function [4–6], cartilage integrity [7–9], mental health measures [10–12], as well as a host of other factors have been observed [13, 14]. In addition, regular aerobic exercise can help to both prevent and treat various disease conditions including cardiovascular disease [15–18], diabetes [19–23], psoriasis [24, 25], and many others [26–29]. Given these positive effects of exercise and the fact that aerobic exercise has inherently low risks for most healthy individuals, it is widely recommended as part of a healthy lifestyle [24, 30–32]. In addition, changes in exercise and diet are standard treatment recommendations to combat the obesity rates increasing in much of the world [33].

Despite the popularity of regular exercise (approximately 50% of adults in the United States meet the guidelines for aerobic exercise set by the Center for Disease Control [34]), physiological variation in how individuals respond to exercise is poorly understood [35]. Variation between individuals is seen in numerous exercise response measures including changes in body weight, cognitive function, metabolic activity, gene expression, and epigenetic marks, all observed in response to aerobic activity [36–42]. Illustrating an extreme case, one recent study has demonstrated that there are individuals who show no or negligible metabolic improvements following an exercise regime [43]. Given the popularity of exercise for its health benefits, it is imperative that we improve our understanding of the genetic architecture underlying exercise response.

Recently, the fruit fly *Drosophila melanogaster* has emerged as a promising model system for exercise studies [44–47]. While studies in humans have been crucial for uncovering how exercise affects human health, humans are not ideal for genetic studies as environmental factors and genetic background are difficult to control. The rodent models can overcome these shortcomings, but rodents are costly to maintain in the numbers required for rigorous genome-wide association studies. Drosophila can be maintained in large isogenic populations in tightly controlled environmental conditions at modest costs [48]. Furthermore, Drosophila is a well-established genetics model, with an abundance of resources including large mutant collections and populations optimized for genome-wide association studies. For example, the Drosophila Genetics Reference Panel 2 (DGRP2) is a collection of 200 genetically diverse inbred lines selected for quantitative genetic studies that have been fully sequenced [49, 50]. Finally, an estimated 75% of human disease genes have a functional Drosophila homolog [51, 52], ensuring that results from Drosophila exercise research can inform human studies. Thus, Drosophila exercise research is ideally positioned to make contributions to the field of exercise genetics by complementing the research carried out in mammalian systems.

While exercise research in Drosophila is a relatively young field, several Drosophila studies have demonstrated that exercising Drosophila can precipitate both behavioral and physiological responses [44–47]. Most of these studies use the Power Tower, an apparatus that induces exercise by repeatedly dropping the fly enclosures, thus causing the flies to fall to the bottom and prompting them to climb up due to their inherent negative geotaxis [47]. Recently, a second Drosophila exercise system, the TreadWheel, has been characterized [46]. This system uses slow rotations of the fly enclosures to induce exercise, again taking advantage of the negative geotaxis response of the animals. Studies with both exercise machines have successfully increased the animals’ activity levels and impacted outcome measures such as metabolite levels, lifespan, gene expression levels, and climbing ability [44–47]. Thus, these studies have established Drosophila as a model for exercise research.

While Drosophila has great promise as a model organism for exercise studies, the system currently suffers from one major shortcoming: To date, no method exists to quantify the amount of exercise performed by the animals. Such a quantification system is necessary to allow standardization of exercise amount between different animals, genotypes, and sexes. In mammalian exercise models, for example, the maximal oxygen consumption measure VO_2_ max can be used to standardize exercise interventions [53]. VO_2_ max measures the maximum (max) of oxygen (O_2_) consumed by a test subject, human or animal, as volume (V) per minute. Typically, exercise is then standardized as a percentage of VO_2_ max. To ensure equivalent levels of effort among individuals despite different baseline physical fitness, each subject works out at 70% of their VO_2_ max [54], thus allowing for standardization. As it is essential for exercise studies that all subjects experience the same level of exercise, a measure that allows for the standardization of exercise regimes is needed urgently for Drosophila.

Our laboratory has developed a novel Drosophila exercise apparatus, the Rotating Exercise Quantification System (REQS), which allows the experimenter to measure the amount of exercise performed by the animals. This unit uses rotations to induce exercise in the same manner as the TreadWheel, and it is capable of recording the activity levels of the flies during the exercise regime. Thus, the system allows for the real-time quantification of activity levels during exercise in Drosophila. Here, we validate the use of the REQS by quantifying the activity levels of four genetically distinct fly strains from the DGRP2, both with the REQS and independently by video monitoring. We find that the REQS can reliably measure activity during exercise and that these results correlate well with independent video monitoring. We also demonstrate the ability of the REQS in detecting differences in exercise behavior between strains and illustrate how it can be used to compare the impact of different exercise regimes. Our findings validate the methodology used by the REQS and demonstrate its utility as an additional tool for Drosophila exercise studies.

## Materials and methods

### REQS

**Fig 1:**
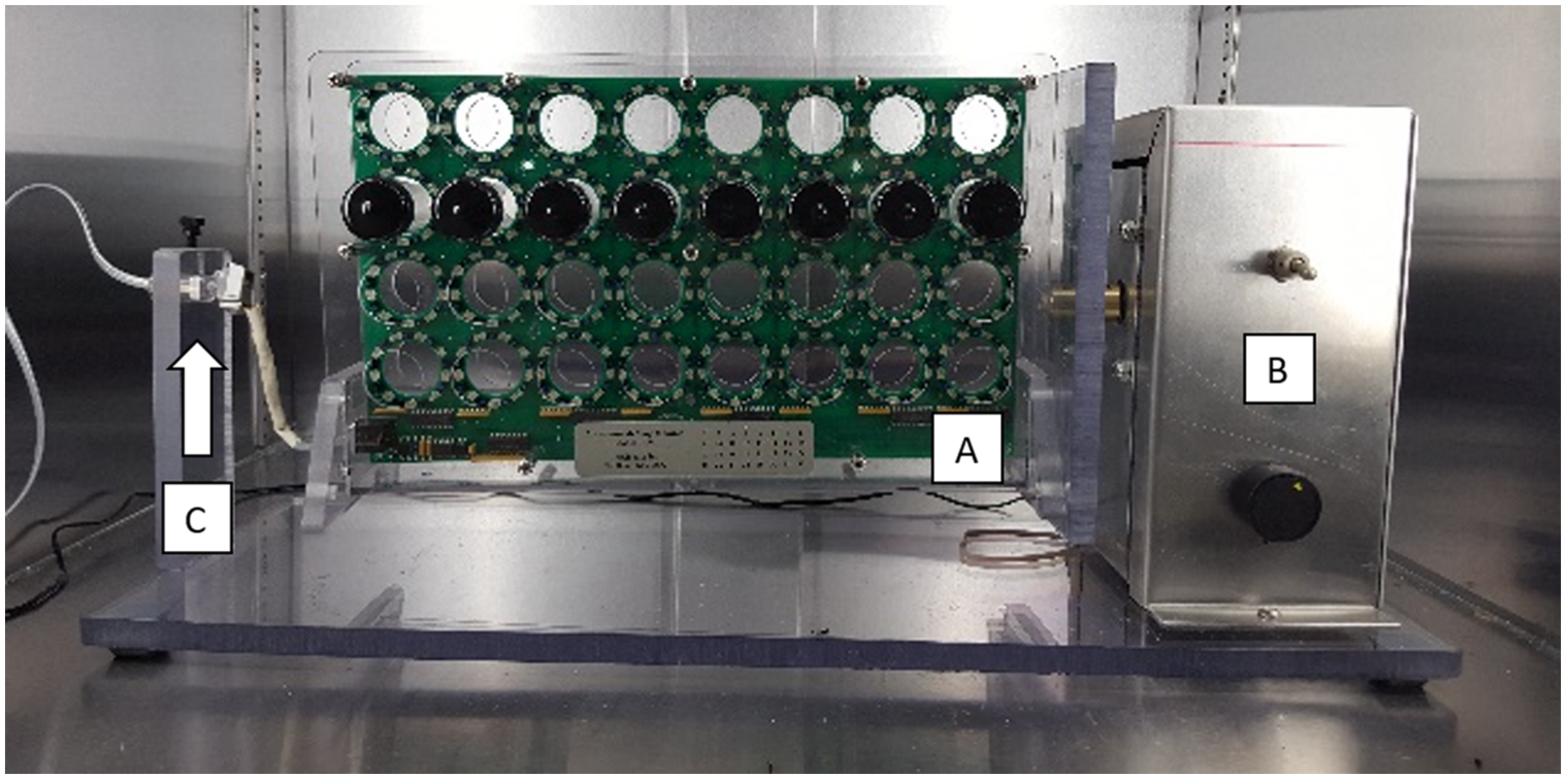
The Rotating Exercise Quantification System (REQS). The unit features the Locomotor Activity Monitor 25 High Sensitivity Unit (A) attached to a side plate which is mounted to a custom adjustable rotator (B) with a power switch and an RPM adjustment knob. In order to avoid tangling of the data cable and to compensate for the rotation, an internally rotating cord unit is placed at C (arrow) to rotate with the LAM25H.

The REQS was developed by the Riddle Laboratory in collaboration with the UAB Research Machine Shop. The unit consists of a Locomotor Activity Monitor (LAM25H) from Trikinetics (www.trikinetics.com) mounted to a speed-adjustable rotating arm (Fig 1, S1 and S2 Figs). The LAM25H measures activity levels by recording the number of laser beam crossings through the center of each vial. The high-resolution unit is capable of tracking multiple individuals crossing the beams at the same time. Data are transmitted through a standard telephone cable from the REQS to a USB interface and then to a computer. In order for the data cable to accommodate the rotation of the device, we have mounted a rotating phone jack (Softalk 21002 Phone Cord Detangler Clear/Black Landline Telephone Accessory) to the data cable end of the REQS, which allows a short data cable to rotate with the machine in order to avoid tangling of the cords. The frequency of data logs can be manually set in the DAMsystem software supplied with the LAM25H, and other accessory options such as recording frequency can be modified by referring to the DAMsystem user guide (www.trikinetics.com).

### Experimental conditions

DGRP2 lines 48, 73, 100, and 181 (Bloomington stock numbers 55016, 28131, 55017, 28151) were used for the REQS characterization study. Three additional lines were used for the exercise regime comparison study (DGRP2-307, 315, 852, Bloomington stock numbers 25179, 25181, 25209).

All flies used in this study were bred and raised in a 12-hour light/dark cycle (8AM– 8PM light) incubator set at 25°C with a humidity of ~70%. Flies were fed a cornmeal/yeast/molasses/agar diet [55]. Flies were between three and seven days old at the time of exercise/activity monitoring. Exercise measurements were taken every day between 11AM and 2PM. Drosophila strains show significant variation in their activity levels and sleep-wake patterns due to genotypic differences. The time frame from 11AM to 2PM was chosen as Drosophila strains inherently tend to show low levels of activity at this time [56–61]. 100 virgin male and 100 virgin female flies of a single genotype were anesthetized with CO_2_ and loaded into exercise vials (10 flies per vial for 10 vials of each sex, 20 vials total per genotype). At 9AM vials were loaded onto the REQS in alternating order of sex (male, female, male, female, etc.). Vials were secured using 17/18-gauge O-rings on the capped sides and with rubber bands on the foam-topped ends to ensure that no movement was possible for the vials during rotation. The machine was loaded into the incubator and positioned such that the vials were oriented vertically (black caps down); this conformation is equivalent to the conditions in which flies are usually kept in the laboratory, in upright vials, thus allowing us to estimate baseline activity levels. From 11AM to 12PM basal activity levels were recorded. At 12PM the rotational unit was set to 4RPM, switched on to induce exercise, and exercise activity levels were recorded from 12-2PM. The same protocol was followed for the experiment comparing exercise regimes, except that basal activity was not recorded. The DAM308 software package that accompanies the LAM25H was used for recording activity levels during the exercise regime (www.trikinetics.com). Activity levels were recorded in five minute intervals for one (basal activity) or two hours (induced activity).

### Video monitoring

Individual flies were loaded onto petri dishes containing agar (9mm diameter, 1% agar) through mouth aspiration. Following a five-minute acclimatization period, basal activity was video-recorded for 10 seconds on a 30FPS camera (Samsung Galaxy S6, 1080P) mounted directly above the agar plate. Immediately following the basal activity recording, the plate was disturbed through tapping, and a 10-second video of induced activity was recorded. 300 frames of each video were analyzed. A total of 10 individuals of each sex were studied per genotype for this experiment. The videos were analyzed using the Tracker Video Analysis and Modeling Tool for Physics Education software (version 4.93, http://physlets.org/tracker/). The auto-tracker feature of the software was used to determine the velocity of the flies through the experiment. An area under the curve calculation was then used to calculate the total distance traveled by the fly.

### Exercise regimes

All experiments except for the exercise regime comparison were performed with two-hour continuous exercise of the animals. When comparing exercise activity levels between different regimes, we compared the two-hour continuous exercise regime to an interval method. The interval exercise regime consisted of a 30-minute exercise period, followed by a 10-minute rest period, a 20-minute exercise period, a 10-minute rest period, followed by a final 10-minute exercise period.

### Statistical analysis

All statistical analyses were performed using either SAS 9.3 or Rstudio [62] (version 3.2.3 “Wooden Christmas Tree”). Analysis of variance (ANOVA) was initially carried out including all possible interaction effects. As there was no significant vial effect, vial was not included in the final model. After the initial analysis, interactions not meeting the significance threshold (p>0.05) were removed from the model, the ANOVA was run a second time, and results from this second analysis are reported.

## Results

### The REQS quantifies exercise from rotation induced activity by recording of laser crossings

We previously demonstrated that rotation can be used to induce exercise in Drosophila which lead to physiological and gene expression changes similar to those seen in other systems [46]. To facilitate the use of Drosophila for the study of exercise genetics, we have developed the REQS, an exercise apparatus that can record activity levels of exercising flies (Fig 1). The unit consists of a LAM25H activity monitor from Trikinetics mounted to a rotating arm. It induces exercise by exploiting negative geotaxis similar to the Treadwheel [46], while recording activity levels as animals cross the laser beams of the activity monitor unit. All components used are commercially available, and the unit can be constructed easily by a departmental machine shop or similar.

**Fig 2:**
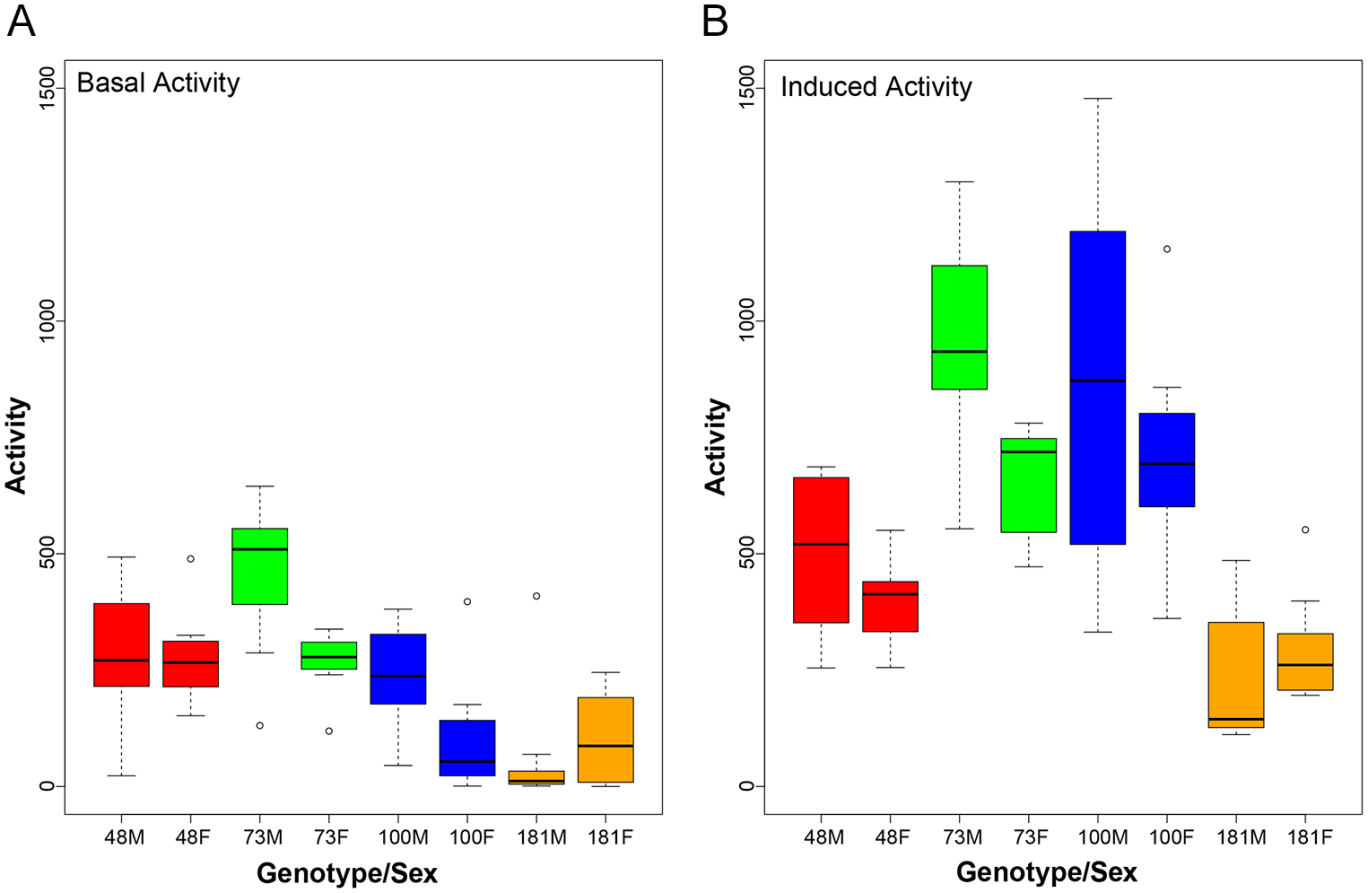
Rotation on the REQS induces exercise as measured by increased activity levels. Activity levels were assessed as the number of laser crossings for four DGRP2 lines separately from male and female flies in an unstimulated environment **(A)** and with rotation **(B)**. 10 vials with 10 flies each were examined for each genotype-sex combination. The Y-axis shows activity levels as cumulative number of laser crossings detected per vial per one hour, while the X-axis shows the genotype and sex of the flies (M: male; F: female, with the number referring to the DGRP2 line). The box indicates the interquartile range, while the lines above and below indicate the maximum and minimum respectively. The middle line indicates the median and dots represent outliers.

First, we confirmed that the REQS is indeed able to measure activity levels while inducing the flies to exercise. Basal activity was measured with the REQS without rotation, with the vials in vertical position, the position that flies are typically stored in the laboratory setting. Then, exercise was induced by rotation, and activity levels were measured again. Both, basal activity and induced exercise activity levels were measured in male and female flies from four strains of the DGRP2 collection (DGRP-48, 73, 100, and 181). We are able to detect significantly higher activity levels in the exercising flies compared to their basal activity levels for all lines (treatment effect; p<0.0001; Fig 2, Table 1). For example, line 100 showed an average of 165 laser crossings per hour, which is increased by nearly 5-fold (790 laser crossings per hour) through the rotation-induced exercise provided by the REQS. Activity levels measured with rotation (induced) are significantly higher than basal activity levels for all four genotypes (Tukey’s host-hoc test; line 48 –p = 0.051; line 73 –p<0.0001; line 100 –p<0.0001; line 181 –p = 0.0130), for lines 73 and 100 even when both sexes are considered separately. Consistent with our earlier studies using the Treadwheel [46], we detect impacts of sex and genotype on activity levels (Table 1; see below for details) and demonstrate that, like the Treadwheel, the REQS is able to induce exercise, i.e. an increase in activity levels above baseline. In addition to inducing exercise, however, the REQS is able to measure activity while the activity monitor unit is rotating. Thus, the REQS can provide a standard for measuring exercise which was previously unavailable for Drosophila.

**Table 1:** The REQS induces increased activity.

Source	DF	SS	Mean Square	P
**Line**	3	3928772.413	1309590.804	<.0001
**Sex**	1	385238.756	385238.756	0.0001
**Treatment**	1	4996369.225	4996369.225	<.0001
Line*Sex	3	486646.631	162215.544	0.0004
Line*Treatment	3	1488478.037	496159.346	<.0001

Analysis of variance reveals a strong treatment effect when comparing basal and induced activity levels, indicating that the REQS is capable of stimulating activity in the animals.

DF–degrees of freedom; SS–sum of squares (Type 1); P–P Value.

### Correlation analysis and video-tracking confirm the results obtained by the REQS

Measuring animal activity via monitors such as the LAM25H is a well-established practice [63–65]. To ensure that rotation does not interfere with the function of the activity monitor, we investigated basal and induced activity measures in a sample of 130 different fly lines. In the majority of cases, we find that the induced activity measures are higher than the basal activity measures and there is a high positive correlation between basal and induced activity. The Pearson’s correlation coefficient between basal and induced activity levels is 0.51 (p = 1.76e-10) in females and 0.34 (p = 6.535e-05) in males. These findings indicate that the activity monitor is functioning correctly while rotating.

**Fig 3:**
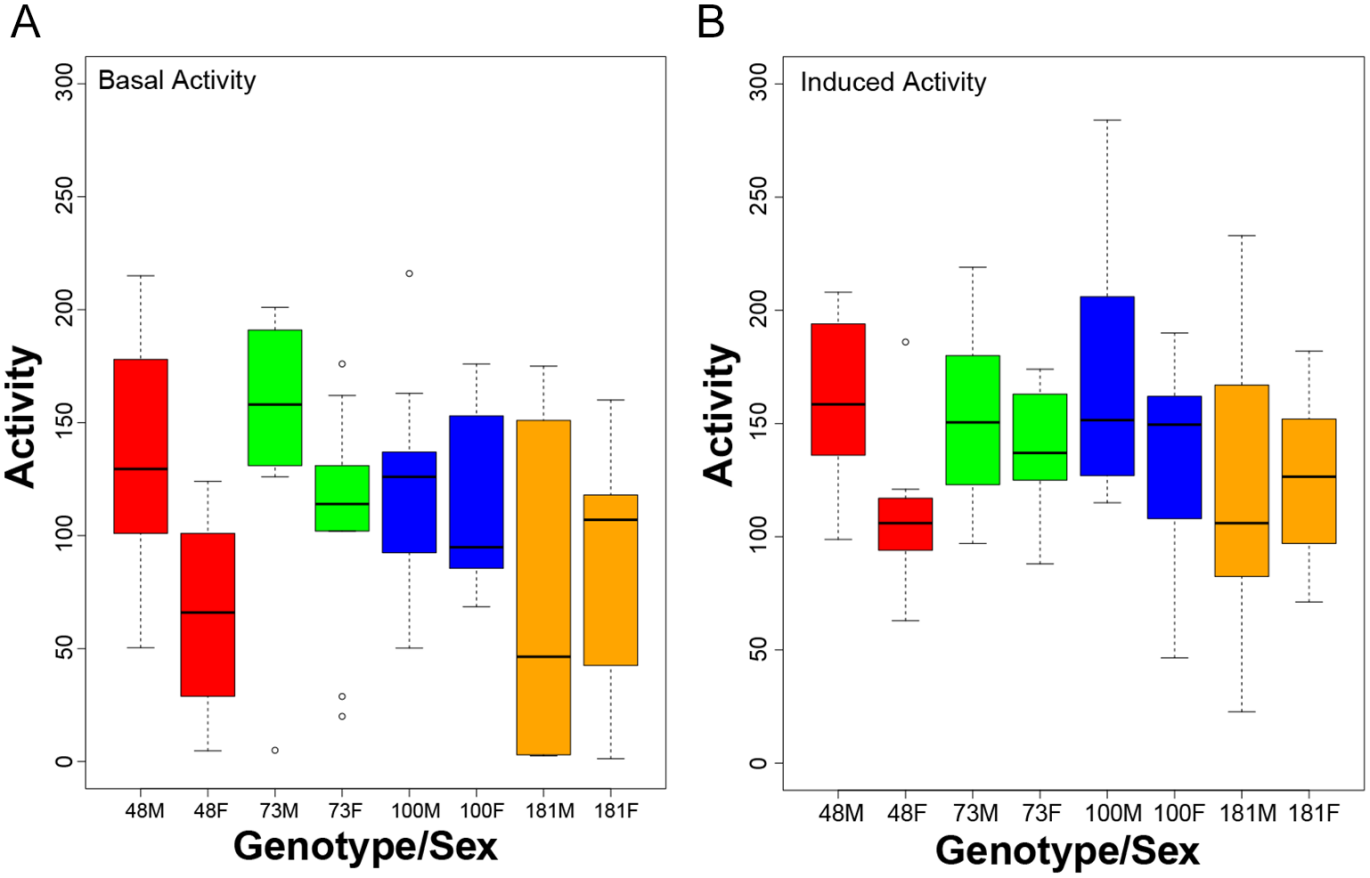
Activity measurements by video-tracking detects variation in fly activity correlate well with the activity levels measured by the REQS. 10 male (M) and female flies (F) from each line were individually video recorded at basal (B) and induced (I) states, and their distance traveled was analyzed. The Y-axis shows distance traveled (mm), and the X-axis shows the genotype and sex of the flies. Both basal and induced activity levels were recorded for 10 seconds. The box indicates the interquartile range, while the lines above and below indicate the maximum and minimum respectively. The middle line indicates the median and dots represent outliers.

To further confirm the activity measurements taken by the REQS, we used video-tracking analysis to assay the activity of the same fly strains used in the REQS analysis above. While this method of inducing activity above baseline levels is very different from the rotation used by the REQS, we expect that in general fly lines with high activity detected by activity monitors will also show high levels of activity in other activity assays and vice versa for low activity lines. For video-tracking, individual flies were placed in a petri dish containing a layer of agar. After acclimatization, the flies were video-recorded with and without stimulation with a camera positioned above the petri dish. From the recordings, activity measures were extracted (Fig 3). Similar to the activity levels measured by the REQS, there is considerable variation between the different genotypes and sexes in both basal and induced activity, but induced activity is consistently higher than basal activity (Treatment effect, p = 0.0056, Table 2). Despite the fact that the activity measures used are very different from those of the REQS, we found that both measurement systems correlate well with each other (compare Fig 2B to Fig 3; Table 3.) In particular, there was a perfect correlation between the ranked genotype-specific activity levels for basal activity, and the rankings only differed slightly for the induced activity levels (the two top-ranking, most active lines were switched). This discrepancy is most likely due to the difference in methodology: for the REQS activity was induced through continuous stimulation by slow rotation over a long time period, while for the video-monitoring it was induced by mechanical tapping and only a short burst of activity was assayed. Given that the results between the video-tracking and the REQS parallel each other, and the fact that basal and induced REQS measures show a high degree of correlation, these data indicate that the REQS is reliably monitoring animal activity monitoring during rotation.

**Table 2:** Activity levels as measured by video tracking depend on treatment, sex, and genotype.

Source	DF	SS	Mean Square	P
**Genotype**	3	32656.37807	10885.45936	0.0030
**Sex**	1	24045.53814	24045.53814	0.0013
**Treatment**	1	43113.34091	43113.34091	<.0001
Genotype*Sex	3	23008.64492	7669.54831	0.0191

ANOVA table demonstrating that the variability observed in activity levels is influenced by treatment, sex, and genotype.

DF–degrees of freedom; SS–sum of squares (Type 1); P–P Value.

**Table 3:** The REQS measurements and independent video monitoring produce similar rankings of different fly strains by activity levels.

Rank (Activity)	Basal (REQS)	Basal (Video)	Induced (REQS)	Induced (Video)
**1**	73	73	73	100
**2**	48	48	100	73
**3**	100	100	48	48
**4**	181	181	181	181

Each fly strain was assigned a rank based on their mean basal and induced activity levels, as measured either by REQS or video monitoring (Line 48, 73, 100, 181). Ranks were assigned in descending order of activity.

### The REQS can be used for comparing exercise regimes

**Fig 4:**
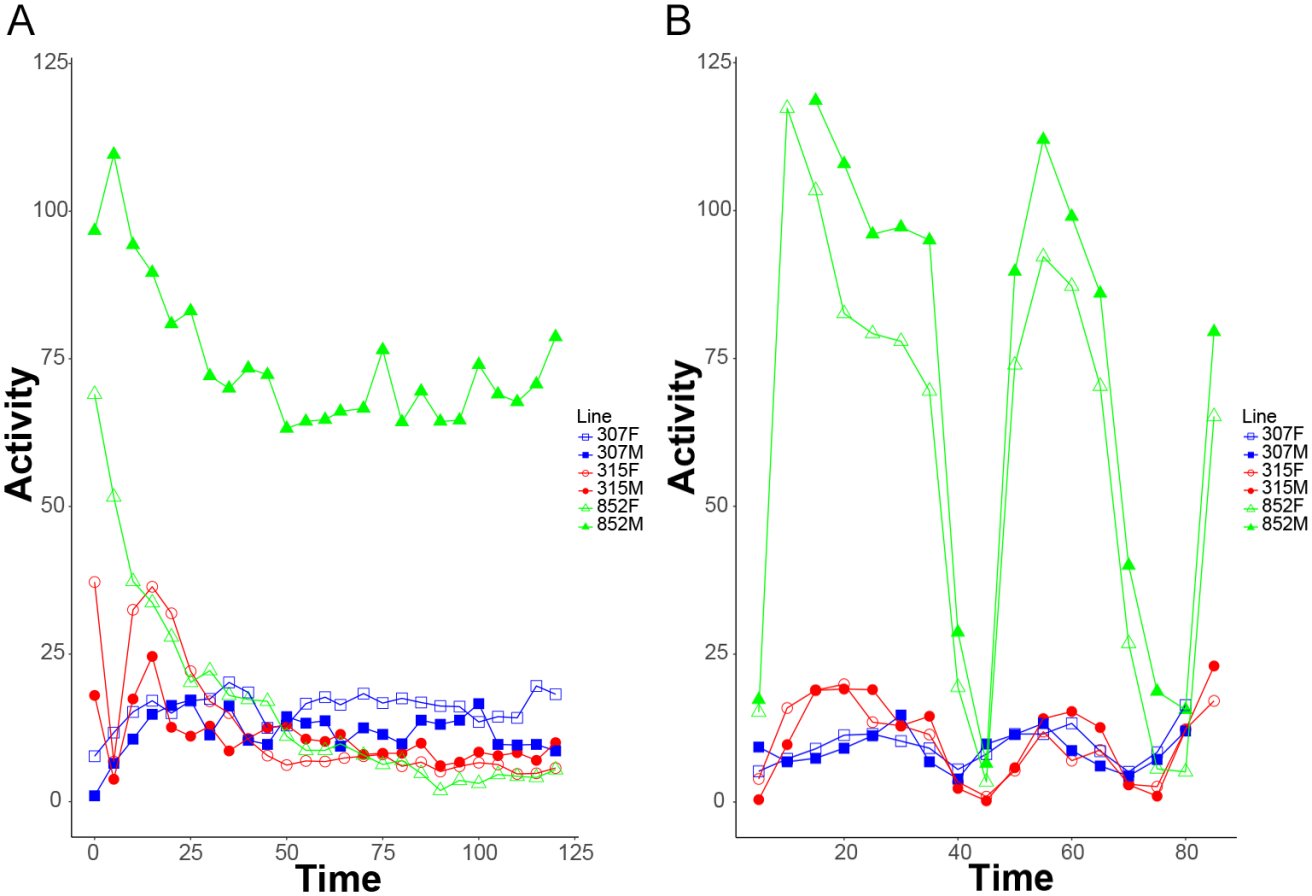
The REQS can detect differences in activity levels between different exercise regimes. Activity of flies from three lines was recording, either during a continuous 2 hour exercise period (A) or during an interval training method (30 minutes exercise, 10 minutes rest, 20 minutes exercise, 10 minutes rest, 10 minute exercise; B). 10 vials with 10 flies per vial were recorded for each genotype and treatment (n = 10). The chart is designated as follows: 307 = blue squares, 315 = red circles, 852 = green triangles, females = open shapes, males = filled shapes. The Y-axis shows mean activity measured as laser crossings per 5-minute interval, and the X-axis shows time in minutes.

As exercise research progresses in Drosophila, there is a need to develop more complex exercise regimes and to compare the activity levels induced by the different regimes. To illustrate how the REQS can be used to evaluate the difference between exercise regimes, we compared a continuous exercise regime to an interval training regime. The continuous exercise regime was induced by two hours of continuous rotation on the REQS, while the interval regime consisted of 30-, 20-, and 10-minute exercise bouts induced by rotation separated from each other by 10-minute rest periods without rotation. To compare the activity levels induced by these two exercise regimes, we examined the number of laser crossings in consecutive 5-minute intervals while the animals exercised. Note, compared to Fig 2, these activity measures are lower due to the shorter time period analyzed (5 minutes versus 1 hour). We find that Drosophila activity levels vary greatly between the two types of exercise regimes examined (Fig 4). Flies experiencing the continuous exercise regime exhibit a slow decline of activity over time, with the rate and magnitude of the decline being genotype-dependent (Fig 4A). The interval training showed that close to maximum activity levels can be recovered in the flies if they are given 10-minute rest periods (Fig 4B). In addition, the results from the interval exercise regime demonstrate that rotation is truly inducing activity as during the rest periods activity declines sharply regardless of line (Fig 4B). Thus, these data confirm that vial rotation does indeed induce exercise and illustrate how the REQS can be used to compare the impact of multiple different exercise regimes.

### The REQS detects genotype- and sex-specific differences in activity levels

In addition to demonstrating that the REQS is indeed able to measure increased activity in response to rotation, the data collected here also provide insights into the sources of variation in activity levels. As illustrated in Fig 2, environmental stimuli such as rotation have profound influences on activity levels, as shown by the increase of activity in the presence of the rotational stimulus observed for all genotypes (Table 1, p<0.0001). In addition, the variability observed between the different sets of flies suggests that activity levels of flies during exercise are genotype- and sex-dependent. Comparing activity levels between groups using analysis of variance, we find that there are significant differences in both basal and induced fly activity levels based on both genotype (p<0.0001 for both) and sex (p = 0.0044, p = 0.0069) individually (Figs 2 and 3, Table 4). Moreover, there was a significant interaction effect between line and sex affecting both basal and induced (p = 0.0106, p = 0.0294) fly activity levels (Figs 2 and 3, Table 4). Thus, the results showed that Drosophila exercise levels are dependent on both the genetic backgrounds and sex, as well as on their interaction.

**Table 4:** Activity levels depend on treatment, sex, genotype, and the interaction between sex and genotype.

**A. Basal activity levels**				
**Source**	**DF**	**Type I SS**	**Mean Square**	**P**
**Line**	3	973803.7375	324601.2458	<.0001
**Sex**	1	114080.5125	114080.5125	0.0044
Line*Sex	3	159210.0375	53070.0125	0.0106
**B. Induced activity levels**				
**Source**	**DF**	**Type I SS**	**Mean Square**	**P**
**Line**	3	4443446.712	1481148.904	<.0001
**Sex**	1	291611.250	291611.250	0.0069
Line*Sex	3	358981.525	119660.508	0.0294

ANOVA table demonstrating that the variability observed in activity levels is influenced by sex, genotype (line), and the interaction between sex and genotype.

DF–degrees of freedom; SS–sum of squares (Type 1); P–P Value.

## Discussion

The results of this study demonstrate that we have successfully implemented a system that is capable of precisely measuring fly activity while inducing increased physical activity, i.e. exercise. The REQS relies on an activity monitoring unit (LAM25H) which is part of the Drosophila Activity Monitoring (DAM) series that is extensively used in Drosophila research and is commercially available [47, 65–67]. Its small size ensures that the REQS fits into a standard Drosophila incubator, and its relatively simple construction makes it possible for researchers to build their own REQS with the help of a machine shop or similar facility. To demonstrate the reliability of this system, the REQS activity measurements were compared to video-tracking data from the same lines. Despite significant differences in methodology, the data from the REQS and video monitoring correspond well to each other, indicating that the REQS accurately records fly activity. The REQS characterization experiments described here demonstrate that it can induce exercise reliably in Drosophila through rotational stimulation of negative geotaxis. These results provide additional validation for the TreadWheel rotational exercise system [46] and confirm that rotational exercise, either via the REQS or the Treadwheel, is a promising tool for the study of exercise in Drosophila.

The REQS presents the first system that allows for the quantification of exercise in Drosophila. While ideally, the distance traveled by each fly would be tracked, this approach is currently not feasible, and the REQS offers a reliable alternative measurement of activity during Drosophila exercise regimes. Thus, for the first time, exercise biologists working with Drosophila will be able to standardize exercise regimes and adjust them between different genotypes to ensure all animals experience the same level of exercise. The ability to estimate the amount of “work done” by the animals–even by a proxy such as beam crossings—is crucial, as our experiments and those of others [68–71] demonstrate that despite the same stimulus, animals can perform vastly different amounts of exercise/work. For example, despite identical stimulation by rotation, the four different Drosophila strains investigated here vary by a factor of four in the amount of exercise they perform (Fig 2). With the data provided by the REQS, this variability can now be taken into account to when interpreting outcome measures such as changes in body weight or physical fitness. Thus, exercise regimes can be adjusted for each animal group under study to ensure that an equivalent level of exercise is performed independent of factors such as sex, age, and genotype. With the REQS providing a measurement of exercise level, exercise studies in Drosophila can now be designed in a similar manner as mammalian studies that have a variety of tools to standardize exercise treatments.

While Drosophila is a relatively new model organism to be used in exercise research, its utility is growing due to the availability of innovative exercise systems like the PowerTower, which induces high impact exercise, the TreadWheel, which induces lower impact exercise, and now the REQS, which combines low impact exercise induction with exercise quantification. All three of these systems allow for high-throughput exercise studies–the REQS can handle 32 samples—and the short generation time of Drosophila ensure that exploratory exercise studies in Drosophila can be carried out quickly. Established mapping populations such as the DGRP2 and the large number of mutant strains available from community stock centers ensure that Drosophila exercise studies can quickly move from GWAS/QTL analyses to the evaluation of candidate loci. As humans and Drosophila share many basic metabolic and physiological pathways, candidates confirmed in Drosophila should be more promising candidates to evaluate in the costlier mammalian systems. Given the capabilities of the REQS, we expect this system to advance the field of Drosophila exercise genetics by providing a means to standardize exercise amounts, compare exercise regimes, and shedding light on the relationship between exercise quantity and exercise outcome measures.

## Supporting information

S1 Fig REQS front view. A frontal schematic of the REQS.
(TIF)

S2 Fig REQS bottom view. The rotational unit of the REQS is secured to a plexiglass base with screws and bolts.
(TIF)

S1 File Compressed archive of the raw data files. These files contain the data used in the analyses presented.
(ZIP)
