# Supplementary figures and images for: Characterization of the Rotating Exercise Quantification System (REQS), a novel Drosophila exercise quantification apparatus
Source: PLoS One. 2017 Oct 10;12(10):e0185090. doi: 10.1371/journal.pone.0185090 (PMC5634558; doi:10.1371/journal.pone.0185090)

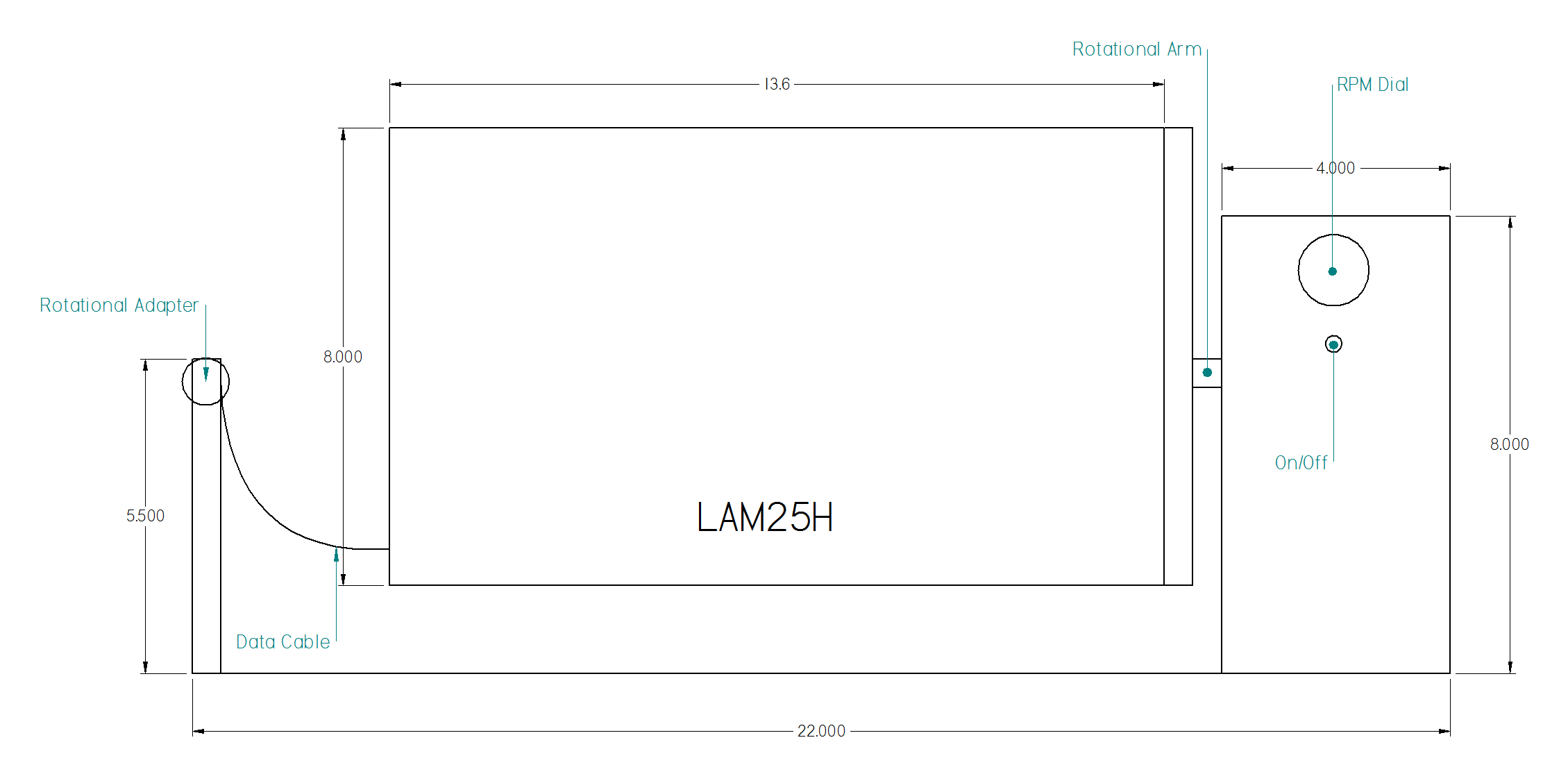

Supplement: S1 Fig — A frontal schematic of the REQS. (TIF) [file pone.0185090.s001.tif]

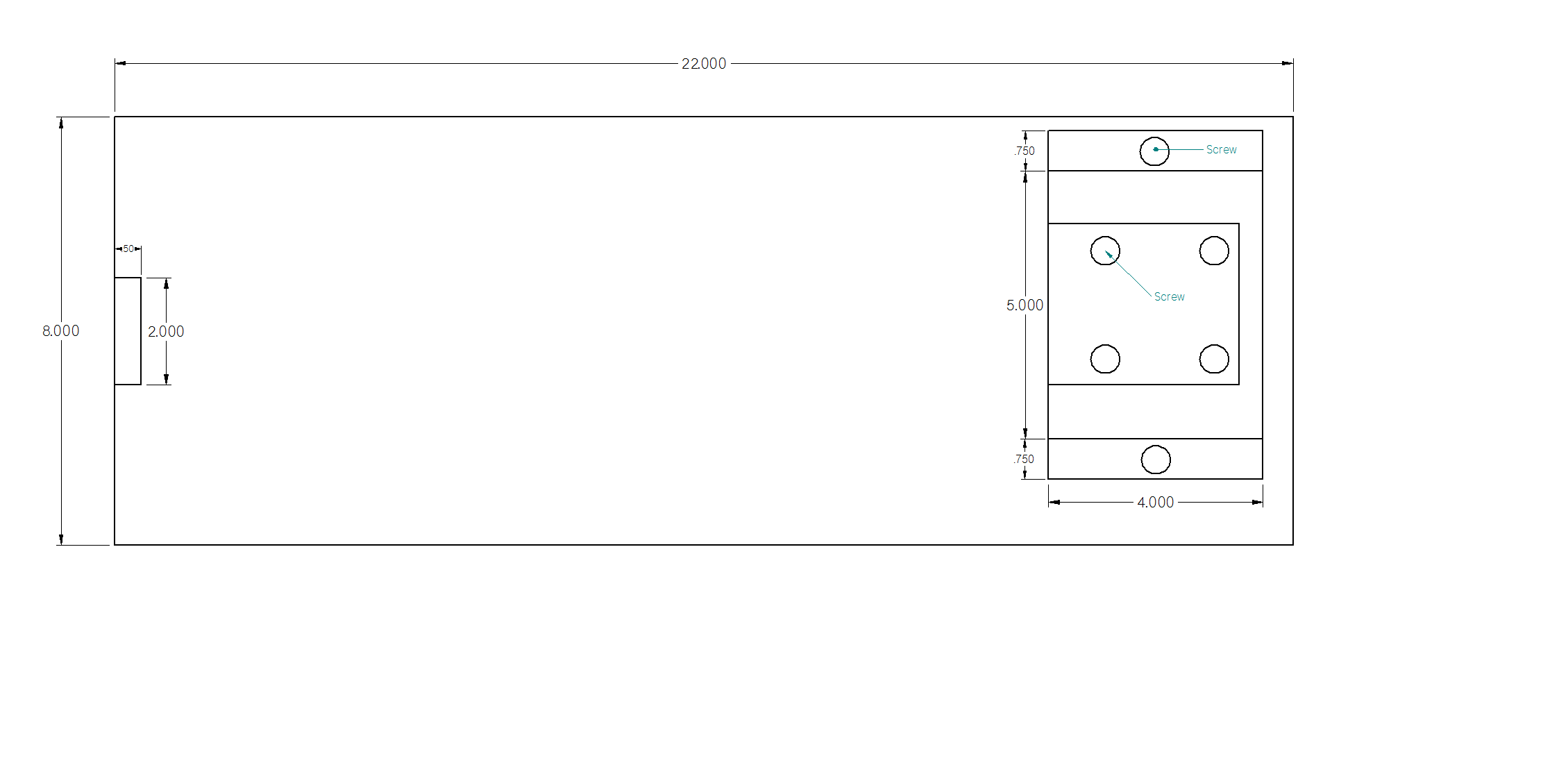

Supplement: S2 Fig — The rotational unit of the REQS is secured to a plexiglass base with screws and bolts. (TIF) [file pone.0185090.s002.tif]
